# A Rare Presentation of Cutaneous Scalp Metastasis From a Malignant Phyllodes Tumor of the Breast: A Case Report and Literature Review

**DOI:** 10.7759/cureus.50009

**Published:** 2023-12-05

**Authors:** Mohammed Alharbi, Laila Ashkar, Atlal Abusanad

**Affiliations:** 1 Faculty of Medicine, King Abdulaziz University Hospital, Jeddah, SAU; 2 Department of Radiology, Faculty of Medicine, King Abdulaziz University, Jeddah, SAU; 3 Department of Internal Medicine, Division of Medical Oncology, King Abdulaziz University Hospital, Jeddah, SAU

**Keywords:** metastasis, scalp, cutaneous metastasis, primary breast malignancy, breast neoplasm, phyllodes tumor

## Abstract

This report presents a unique case of a 56-year-old female diagnosed with a malignant phyllodes tumor (PT). Following a modified radical mastectomy, the patient exhibited metastasis to the lungs, bones, and, uncommonly, the scalp. Despite treatment interventions, including chemotherapy and radiotherapy, the patient’s condition progressed, underscoring the aggressive nature of malignant PTs. This case emphasizes the importance of recognizing unusual metastatic sites and the challenges in managing such aggressive tumors.

## Introduction

Phyllodes tumors (PTs), previously known as cystosarcoma phyllodes, are rare fibroepithelial breast tumors. Originating from the periductal stroma, they consist of both epithelial and stromal elements of the breast, excluding the ducts and glands. These tumors can exhibit a broad spectrum of biological behaviors, ranging from benign to malignant [1,2]. PTs account for less than 1% of all primary breast tumors [3]. PTs typically present as hard, rapidly enlarging lumps and are commonly identified in women aged between 40 and 50 years [4,5]. PTs are classified into benign, borderline, and malignant based on factors such as tumor edges, mitotic rate, nuclear variability, the extent of stromal growth, and cell density [6]. While a significant proportion of PTs are benign (60-75%), the recorded instances of borderline and malignant PTs account for 15-20% and 10-20% of all PTs, respectively [7]. Malignant PTs exhibit aggressive clinical features characterized by rapid growth and the potential for metastasis. They primarily spread via the hematogenous route, with the lungs and bones being the most frequent metastatic sites [8]. Despite their aggressive nature, PTs can metastasize to any organ. However, a few studies have reported unusual metastatic sites, such as cutaneous spread [8-10].

In particular, PT metastases to the scalp are a very uncommon occurrence. To our knowledge, only one case of PT metastasizing to the scalp has been reported without spreading to the brain tissue or the head and neck region [11]. We present the case of a 56-year-old female with a malignant PT, marked by cutaneous scalp metastasis, accompanied by lung and bone metastasis.

## Case presentation

**Figure 1:**
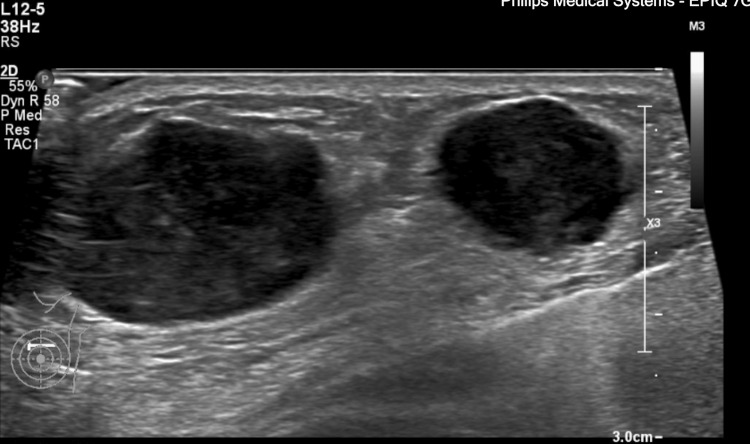
Ultrasound of the left breast with three oval hypoechoic parallel masses, with the largest measuring 2.9 x 1.4 x 2.5 cm.

**Figure 2:**
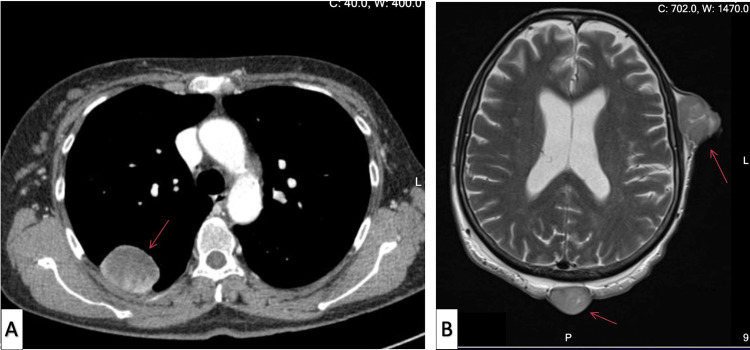
(A) CT of the chest shows a 3 cm mass in the right lower lobe and two nodules in the left lung. (B) MRI of the brain shows multiple well-circumscribed mass lesions overlying the left scalp.

**Figure 3:**
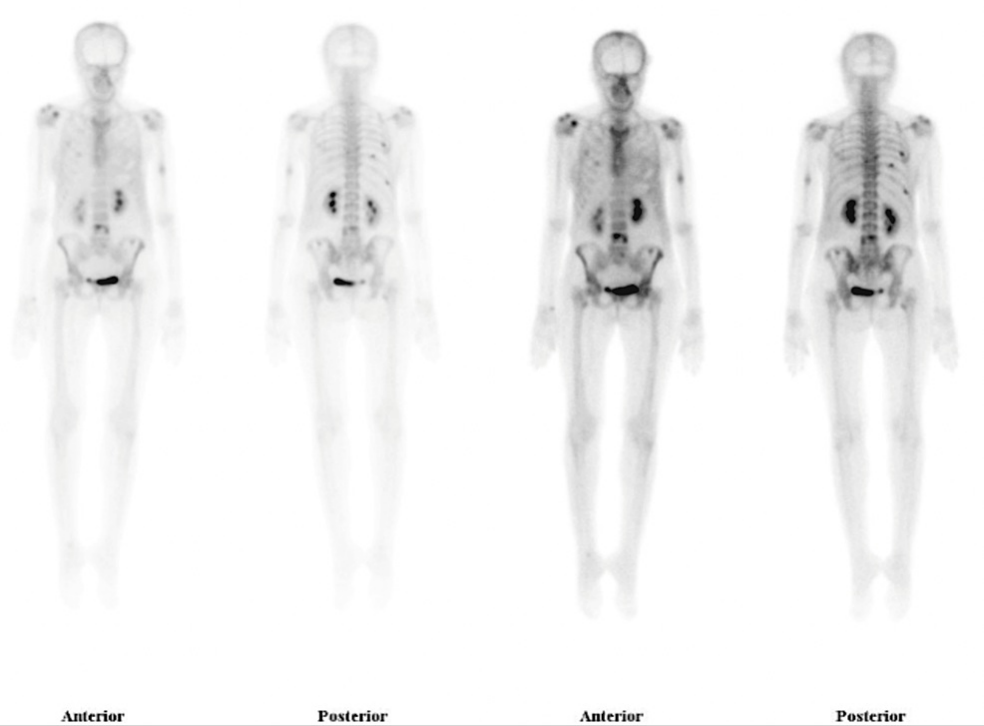
Bone scintigraphy shows increased uptake across the skeleton consistent with metastases.

A 56-year-old female, medically free, initially presented with a lump in her left breast. On subsequent investigations, she was diagnosed with a malignant PT. Consequently, she underwent a modified radical mastectomy. Two months postoperatively, she presented with palpable masses at the surgical site. An ultrasound of the left breast revealed three oval, hypoechoic, parallel masses, with the largest measuring 2.9 x 1.4 x 2.5 cm (Figure 1). A metastatic workup was performed, which included a CT of the chest, abdomen, and pelvis, as well as a bone scan. CT of the chest demonstrated a 3 cm mass in the right lower lobe and two nodules in the left lung, measuring 1.3 cm and 0.3 cm, respectively (Figure 2A). CT of the abdomen and pelvis showed no metastatic lesions. CT of the brain revealed no findings of acute brain insult or obvious space-occupying lesion; however, it highlighted multiple subcutaneous soft tissue masses over the left scalp. Subsequently, an MRI of the brain identified multiple well-circumscribed mass lesions overlying the left scalp, especially along the parietal and occipital bones, with the largest along the left high parietal area, measuring 12 mm in maximum dimensions (Figure 2B). The bone scan showed dominant uptakes in the right mid-humerus, right shoulder, right rib cage, L4, and left iliac bone (Figure 3). Following these findings, the patient underwent chemotherapy and radiotherapy sessions. The chemotherapy regimen was composed of six cycles of IV doxorubicin 60 mg/m² and IV ifosfamide 5,000 mg/m². These agents were administered in combination every 21 days.

**Figure 4:**
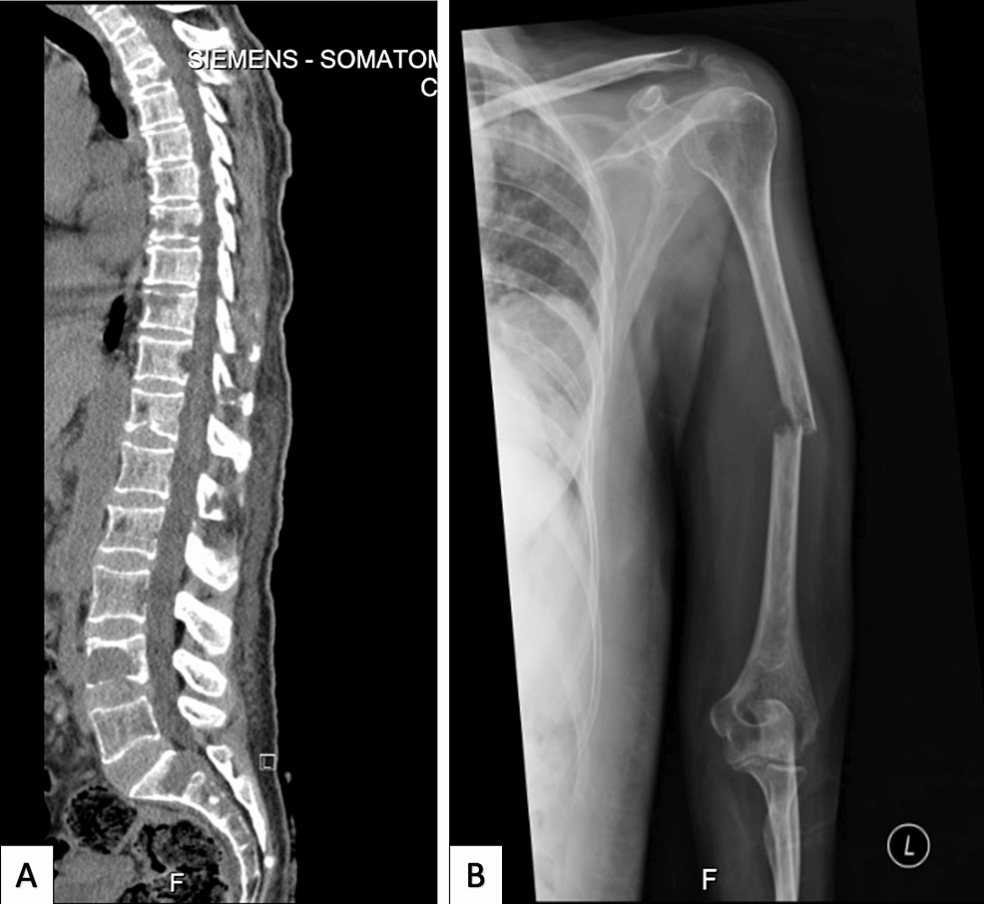
(A) CT of the whole spine shows extensive metastatic disease involving the spine. (B) The left upper limb X-ray shows a humeral shaft transverse fracture.

**Figure 5:**
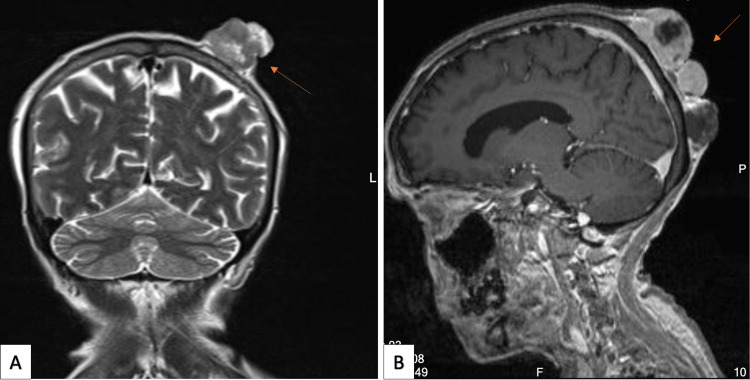
MRI of the brain with progression of the scalp metastatic lesions. (A) Coronal view of the brain. (B) Sagittal view of the brain.

Six months later, she presented with lower back pain radiating to her left lower limb, associated with weakness. She had no history of fecal or urine incontinence but had a history of an incidental fall on her left arm, accompanied by an increase in the size of the previous scalp nodules. On physical examination, the power of the right lower limb was 4/5 while the left lower limb was 3/5 with intact sensation. Her left arm showed swelling, deformity, and tenderness over the shaft of the humerus with limited elbow range of motion due to pain, and the distal neurovascular status was intact. She had three enlarged nodules on the scalp with blood and serous discharge. On investigation, a CT of the whole spine revealed extensive metastatic disease involving the spine (Figure 4A), and an upper limb X-ray showed a humeral shaft transverse fracture (Figure 4B). A follow-up MRI of the brain indicated progression of the scalp metastatic lesions without evidence of brain parenchymal metastasis (Figure 5). The patient subsequently underwent a U-shaped slab procedure on the left arm and was eligible to proceed to palliative radiotherapy.

## Discussion

PTs account for less than 1% of all breast tumors, and the majority are benign [3]. PT consists of epithelial and stromal elements of the breast, organized into leaf-like structures with papillary projections. Local recurrence can occur after initial surgical removal in all types of PTs. PTs generally recur locally within two years [3,12,13]. Some studies have observed that malignant tumors have a shorter local recurrence than benign or borderline tumors [3,14,15]. Based on the tumor grade, a meta-analysis indicated local recurrence rates of 8% for benign tumors, 13% for borderline tumors, and 18% for malignant tumors [16].

Metastases can appear without coexisting local recurrence [3]. According to available data, it can be estimated that the distant metastasis rate for PT varies significantly, ranging from 1.7% to 27% [17,18], with an average of 5.6%, and varies according to tumor grade [8]. While it is extremely rare for benign PTs to metastasize to distant locations, there are rare exceptions, which makes it hard to predict the clinical progression of a PT [12]. Metastasis occurs primarily through the hematogenous route. The most common sites of distant metastasis for PT are the lungs and bones; however, nearly all other organs can be affected. A few studies have reported unusual metastatic sites, such as cutaneous spread [8-10]. We found one case of metastasis of PT to the scalp without spreading to the brain tissue [11]. Our patient showed lung, bone, and scalp metastases with local tumor recurrence.

It is recommended to perform a wide local excision with negative margins of 1 to 2 cm. A simple mastectomy is preferred if achieving negative margins is impossible [19]. As the involvement of axillary lymph nodes by PTs is rarely reported, performing routine axillary lymph node dissection is not recommended [8]. Once metastasis occurs, the prognosis is unfavorable. Multimodal care has been employed for the management of metastatic PT. Standard chemotherapy plays a limited role and is primarily utilized as palliative therapy [20].

## Conclusions

In summary, malignant PT is a rare and aggressive fibroepithelial breast tumor and should not be underestimated, especially in its potential for metastasis. In this case report, we document a particularly rare presentation of cutaneous scalp metastasis originating from a malignant PT of the breast. Notably, while the most common sites for metastasis of malignant PT are the lungs and bones, this case underscores the tumor’s capacity to metastasize to less predictable and unconventional sites, such as the scalp. It highlights the importance of clinicians maintaining a high level of suspicion and a comprehensive approach to monitoring and managing patients with malignant PT, even in the absence of typical metastatic presentations. It is crucial to emphasize molecular research in these tumors, which enhances the understanding and management of malignant PTs. Furthermore, due to the tumor’s rarity, multi-institutional collaboration is essential to establish a representative cohort for valuable investigations.
